# Obstacles and opportunities for care collaboration through the utilisation of a preventive care process for frail older adults: a study protocol for a mixed methods study design

**DOI:** 10.1136/bmjopen-2023-081925

**Published:** 2025-02-10

**Authors:** Martina Karin Boström, Åsa Larsson Ranada, Helle Wijk, Pia Skott, Anette Erichsen, Elisabet Rothenberg

**Affiliations:** 1 Department of Quality Improvement and Leadership, Jönköping Academy For Improvement of Health and Welfare, Jönköping University Hälsohögskolan, Jonkoping, Sweden; 2 Qulturum, Region Jönköpings län, Jönköping, Sweden; 3 Department of Health, Medicine and Caring Sciences, Linkoping University Department of Health Medicine and Caring Sciences, Linkoping, Sweden; 4 University of Gothenburg, Goteborg, Sweden; 5 Institute of Health and Care Science, Sahlgrenska University Hospital, Goteborg, The Sahlgrenska Academy, Sweden; 6 Public Dental Services, Department of Orofacial Medicine, Stockholm University, Stockholm, Sweden; 7 Institutionen för vårdvetenskap och hälsa, University of Gothenburg, Goteborg, Sweden; 8 Health Sciences, Kristianstad University, Kristianstad, Sweden

**Keywords:** Aged, Health policy, Quality in healthcare, Health Services for the Aged, REGISTRIES

## Abstract

**Abstract:**

**Background:**

Frail older adults constitute a significant vulnerable group with complex healthcare needs requiring collaboration between various care providers and professions. Despite this requirement, there are considerable knowledge gaps in how to achieve effective collaboration. Preventive measures such as addressing pressure ulcers, falls, malnutrition and poor oral health often receive low priority compared with treating diseases or injuries. This study aims to enhance our understanding of how care collaboration in the preventive care process (PcP) using the Senior alert (SA) register could improve patient safety and equality of care for frail older adults in Sweden.

**Methods:**

This study, conducted over 4 years, employs an explanatory mixed methods design, divided into three phases. The study protocol comprises the two first phases.

**Phase 1 (quantitative):**

Data from SA (2019–2021) will be analysed to compare municipalities regarding the quality of registration in the PcP. Municipalities with high and low compliance indices will be identified and analysed together with data on demographics, socioeconomics and organisation from Statistics Sweden.

**Phase 2 (qualitative):**

Focus group interviews will be conducted in residential care units in the municipalities identified in phase 1 with interprofessional teams and older persons/next of kin. Additionally, 30 semi-structured individual interviews with residential care managers and other relevant stakeholders will explore obstacles and opportunities for effective care collaboration.

**Discussion:**

We foresee that the results will contribute to the development of a model for effective PcP and care collaboration that can be used to improve patient safety and quality of care for frail older adults. This model can be tested and upscaled to achieve a more effective and equitable healthcare system.

**Ethics and dissemination:**

The study has been approved by the Swedish ethical review authority. Dissemination plans involve publications, data deposition and engagement with healthcare stakeholders to ensure the practical application of the findings.

**STRENGTHS AND LIMITATIONS OF THIS STUDY**

- This national project leverages extensive datasets and uses both qualitative and quantitative methods, allowing for a comprehensive analysis of the complex nature of care collaboration among frail older adults through a novel research approach.
- The critical role of healthcare collaboration emphasises in ensuring coherent care, enhancing patient safety and promoting equitable access across different populations, while investigating barriers and opportunities in care collaboration using the Senior alert’s preventive care process.
- Variability in care collaboration practices across different regions of Sweden presents challenges in achieving consistent outcomes, complicating the interpretation and generalisability of the results.
- The study explores a relatively under-researched area and addresses significant challenges in elderly care, such as the increasing incidence of adverse events among frail older adults, which may complicate the implementation and evaluation of preventive actions.

## Background

 Frailty is a clinical state with an increase in the individual’s vulnerability for developing increased dependency and/or mortality when exposed to a stressor. It is a late-life condition associated with adverse events such as fall, cognitive impairment, disability and hospitalisation. From a healthcare perspective, frail older adults probably constitute the largest vulnerable group in Swedish society, due to a demographic trends (SCB) and high prevalence of multimoribidity (patientregistret) in this age group.^1 2^ Due to their complex healthcare needs, they are dependent on well-working collaboration between various care providers and professions (ie, nurses, dietitians, occupational therapists, dentists, assistant nurses, physicians and social service workers), regardless of the administrative level of their care.^3^ However, the process of care collaboration between different care providers, and healthcare and social service professionals, within or between organisations, is complex and therefore does not always work as intended. Moreover, low staff continuity and inadequate access to staff with appropriate skills make the provision of quality elderly care challenging.^4 5^ Actions to prevent, common adverse events are often overlooked and given low priority compared with the treatment of diseases or injuries.^6 7^ Considering these substantial challenges, we will study care collaboration based on the preventive care process (PcP) in the qualitative register senior alert (SA), one of Sweden’s largest National quality registries, used in 99% of 290 Swedish municipalities and in 67% of the 21 county councils, with about 100 000 unique older individuals risk assessed each year. SA focuses proactively on the common risk areas of malnutrition, pressure ulcers, falls and oral health among people≥65 years of age.

The latest developments in Swedish healthcare indicate an increased awareness of the need for collaboration within and between organisations to better address the complex care needs of older individuals.^8 9^ One approach is to link elderly care with a national knowledge governance initiative, where evidence-based strategies and resource-efficient measures can be developed and implemented.^10^ The lack of contextual analyses, particularly concerning legislation and financing systems, complicates the assessment of the effectiveness and outcomes of collaboration measures.^11 12^ This underscores the existing knowledge gap in relation to older adults with complex care needs and the importance of understanding how collaboration operates in practice in order to facilitate the development and implementation of effective collaboration models.

A greater understanding of the mechanisms that contribute to well-working care collaboration within and between organisations in the PcP, regardless of the level of administration, might increase patient safety,^13 14^ prevent adverse events and promote equality of care for frail older adults^15^ throughout the country. There is no generally accepted terminology in Sweden for the concept of ‘care collaboration’ and a plethora of methods and tools to enable better collaboration can be found in the healthcare sector and/or in research.^16^,^18^ In the literature, the attributes that are associated with collaboration often include concepts such as ‘sharing’, that is, sharing of resources and/or decision‐making. Other attributes of collaboration are ‘teamwork’ and ‘respect’ with regard to improving patient safety and quality of care. In medical and nursing sciences, the concept of collaboration has been highlighted as valuable and might contribute to building trust in the relationships between patients, relatives and the healthcare professions.^19 20^ Positive outcomes of collaboration are quality of care, patient safety, and making it easier for the patient to gain an overview of his/her care process.^21 22^ Previous studies indicate that care collaboration in healthcare seems to be considered highly valuable in principle and that older adults and caregivers want to be actively engaged in a dialogue regarding care,^23^ but that well-working collaboration is difficult to achieve in everyday care.^24^ The difficulties seem to refer to the absence of clear goals or clear evaluation criteria for the care, which contributes to a misconception in collaboration between care providers and organisations.^16^ In research, the value of care collaboration has been questioned.^25 26^ This scepticism is due to the evaluation methods used, where most studies seem to be designed to assess how participants experienced and/or perceived care collaboration.^6^ We believe that different study designs, both qualitative and quantitative, are needed to encompass this complex research area. Therefore, a mixed methods design with exploratory orientation has been chosen. The first quantitative part, based on data from SA and official statistics, might guide more in-depth probing in the qualitative part using individual and focus group interviews. With this design, we aim to achieve a more holistic, reliable and valid understanding of the obstacles to and opportunities for care collaboration within and between care providers.

### Governance of the care of older adults in Sweden

**Figure 1:**
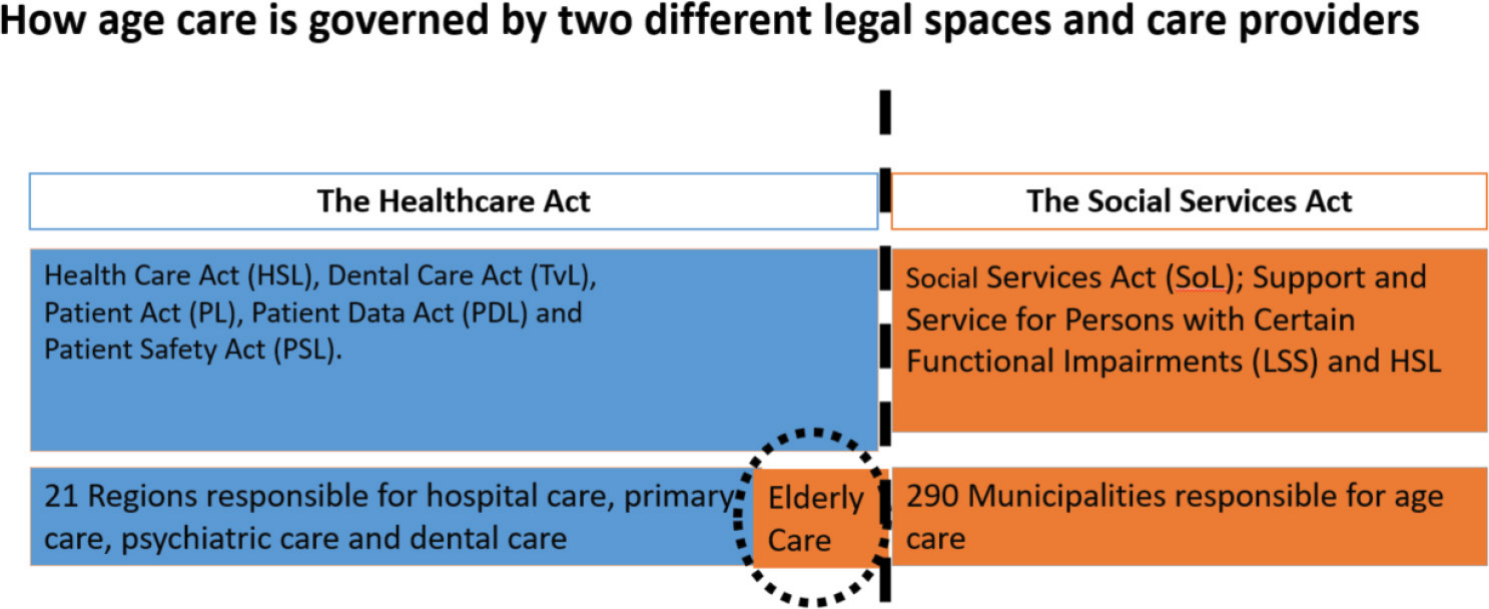
The governance of the health and social care of older adults.

Sweden as a welfare state provides care and a social security system for every individual in the country throughout the entire course of their life. The healthcare system is governed by the democratically elected parliament. The country’s regions and municipalities are responsible for the provision of health and social care services; these are financed by taxes. However, the regions and municipalities have independent powers of taxation, meaning that they tax separately. This can create a financial obstacle to well-functioning care collaboration. Further, the health and social care of older adults is provided by the regions and the municipalities and is governed by two laws: the Social Services Act^27^ (SoL) and the Healthcare Act^28^ (HSL). The regions are governed by the HSL and the municipalities are by the SoL and HSL. SoL is a civil rights law giving the individual specified rights and HSL is a law of obligation addressing the obligations of Swedish care providers. The care of older adults in Sweden is, therefore, complex since it is governed by two legal spaces with different perspectives and different sources of financing; this entails challenges for care collaboration across organisational boundaries (figure 1). In total, 21 regional councils are responsible for the provision of hospital, primary, psychiatric and dental care for the country’s population. The regional councils are responsible for dental care for persons needing special support or dental care as part of disease treatment and/or surgery. In addition to the HSL, the care provider’s obligations are described in the Dental Care Act (TvL), the Patient Act (PL), the Patient Data Act (PDL), and the staff’s obligations are described in the Patient Safety Act (PSL). At a local level, there are 290 municipalities governing residential care facilities and home help care services in accordance with the SoL. Healthcare in residential care facilities and home help care is governed by the HSL. Approximately, 80 000 older adults in Sweden live in municipal residential care facilities^29^ and are permanently in need of care from different care providers.^30 31^

The complexity of elderly care governance and the increased risk of hospitalisation and admission of older adults to residential care facilities create challenges for care collaboration between different care providers. There is, therefore, a need to identify the obstacles to and opportunities for fully using the potential for the collaborative care of frail older adults in the prevention of negative events. Based on the WHO’s definition of prevention,^32^ the importance of preventive measures to reduce, for example, hospital readmissions among frail persons was demonstrated as early as 2001^10 33^ and still poses challenges.^34^ Moreover, a deeper understanding of how the interplay within and between interprofessional teams is perceived within and between different care providers is crucial in order to contribute to patient safety and strengthen the quality of care, regardless of geography, socioeconomics, place of birth, disability, age, gender or ethnic background.^35^

### Senior alert

**Figure 2:**
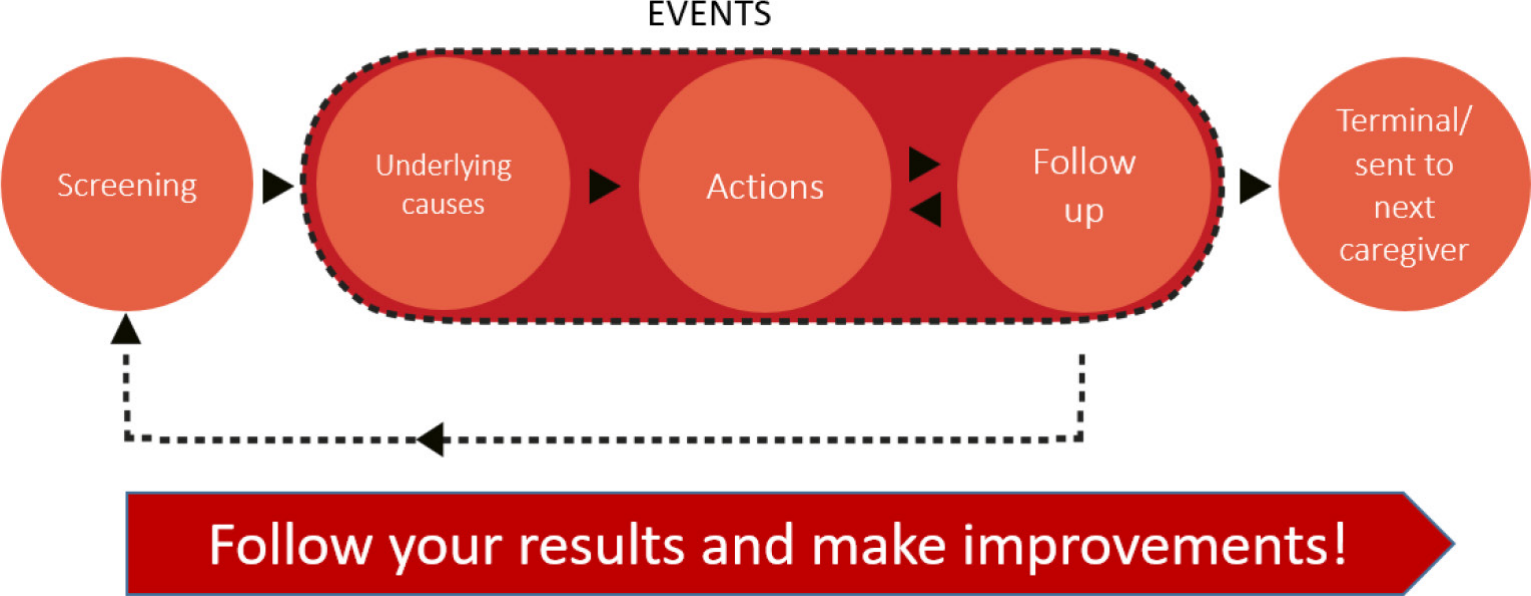
Preventive care process in senior alert.

SA is the largest quality register in Sweden including frail older adults in need of healthcare and long-term care; it is used by 288 of Sweden’s 290 municipalities.^36^ A quality register contains individualised data concerning medical interventions, procedures and outcomes within healthcare provision. Registers are monitored annually and approved for financial support by a National Executive Committee. SA promotes quality improvement through the PcP (figure 2) in which staff screen for the risks of falls, bladder dysfunction, pressure ulcers, malnutrition and poor oral health. After risk assessment, underlying causes, actions and follow-up are implemented and registered in SA, allowing comprehensive monitoring and evaluation at the individual, care unit and societal levels. SA can also be used to achieve other aims, such as providing an integrated and active means of enabling continuous staff learning and for quality improvement in care, as well as for research.

In 2008, the register focused on three areas: malnutrition, pressure ulcers and falls among people≥65 years of age. In 2011, the assessment of oral health was included and in 2014 the assessment of bladder dysfunction was added. In the forthcoming study, the assessment of bladder dysfunction will be excluded since the number of registrations is incomplete; four focus areas will therefore be included. These four risk areas are inter-related; for example, a fall could imply a risk for malnutrition and poor oral health, and poor oral health could exacerbate eating difficulties.^37^ Frail older adults in need of care should therefore undergo risk assessments to ensure that preventive actions are taken, and follow-up is performed to avoid adverse events. Common risks among older people are assessed using validated evidence-based instruments,^38^ Modified Norton scale^39^ Mini Nutritional Assessment^40^ and Revised Oral Assessment Guide-J.^41^

The PcP in SA is based on four steps: (1) **Screening for risk assessment:** aimed at identifying possible risks within the focus areas; (2) **Team-based investigation of the underlying causes:** to allow proper measures to be taken; (3) **Actions to perform interventions/events:** within the focus areas; (4) **Follow-up:** to monitor the effect of the intervention. Based on the outcome of the intervention, decisions may be made regarding the need for new interventions. Complete registration means registration, so to speak compliance, in all four focus areas and in all steps of the process, when a risk has been identified. There is also a need for dialogue and/or practical actions within and/or between care providers. Almost 90 000 risk assessments were performed in residential care facilities in 2020. Risks were identified in at least one of the four focus areas in 92% of cases in residential care facilities and home help care.^42^ However, not all the risks identified led to an intervention, which is in line with previous research concluding that frail persons do not receive planned and performed interventions to the required degree, and SA is therefore not used to its full potential.^43^ A mismatch between identified risks and planned interventions has also been found suggesting that there are flaws in how the PcP is implemented. In summary, despite prioritised initiatives for preventive care for frail older adults in Sweden, evaluation and follow-up of these processes are not carried out to the extent necessary to assess expected outcomes.^4 7^ Understanding how adherence to preventive care can be improved through effective collaboration may help reduce adverse events and improve the quality of care for frail older adults.

### Rationale

The increased demand for the care and support of older adults, particularly frail individuals with complex needs, underscores the necessity of interdisciplinary collaboration to meet these demands. Challenges in collaboration arising from the division of legal frameworks and areas of responsibility highlight the need for a more unified and coordinated approach to elderly care. Concurrently, ongoing initiatives and reforms from organisations such as the Swedish Association of Local Authorities and Regions (SALAR) demonstrate an awareness of these challenges and a commitment to improving collaboration within healthcare. By enhancing the collaboration among different healthcare providers and competencies, we can not only improve the accessibility and efficiency of care for older patients but also reduce the risk of misunderstandings and coordination failures, thereby enhancing patient safety and healthcare quality. Specifically, highlighting SA as a tool for identifying and preventing risks in older patients demonstrates concrete actions taken within the framework of multidisciplinary collaboration to enhance the care of this vulnerable population. On 2 March 2017, the Swedish Government decided to appoint an inquiry chair with the remit of supporting county councils/regions, relevant government agencies and organisations in the coordinated development of modern, equitable, accessible and effective healthcare. The Inquiry chose the name ‘Coordinated development for good quality, local health care’ in Swedish ‘Nära Vård’. In the transition to ‘Nära Vård’, the importance of how collaborative care and a more coherent healthcare system can be organised is central to the success factors that have been described and/or developed.^35^ We intend to study care collaboration in accordance with the PcP in SA, within and between different healthcare organisations, including dental care, and within and between interprofessional teams. When properly used, SA is expected to provide a foundation for the systematic improvement of collaborative care that successfully prevents adverse events and leads to important knowledge within four risk areas in SA. There is also a need to identify the obstacles to and opportunities for fully using SA to achieve a more effective PcP. Moreover, a deeper understanding of these obstacles and opportunities is needed to develop a model for an effective PcP for frail older adults in Sweden. This model can then be tested and upscaled.

### Aim

To explore factors that constitute obstacles and opportunities for care collaboration within and between different care providers in the utilisation of a PcP for frail older adults.

### The research questions will be

1. Are there differences between the quality of registration in the municipalities (QR) (high or low proportion of complete registrations in SA) that can be explained by factors such as demographics, socio-demographics, economy, organisation of elderly care, or political majority?
2. What obstacles to and what support for good collaboration exist within and between different care providers regarding the fulfilment of an effective PcP in SA?

## Methods

### Study design

**Figure 3:**
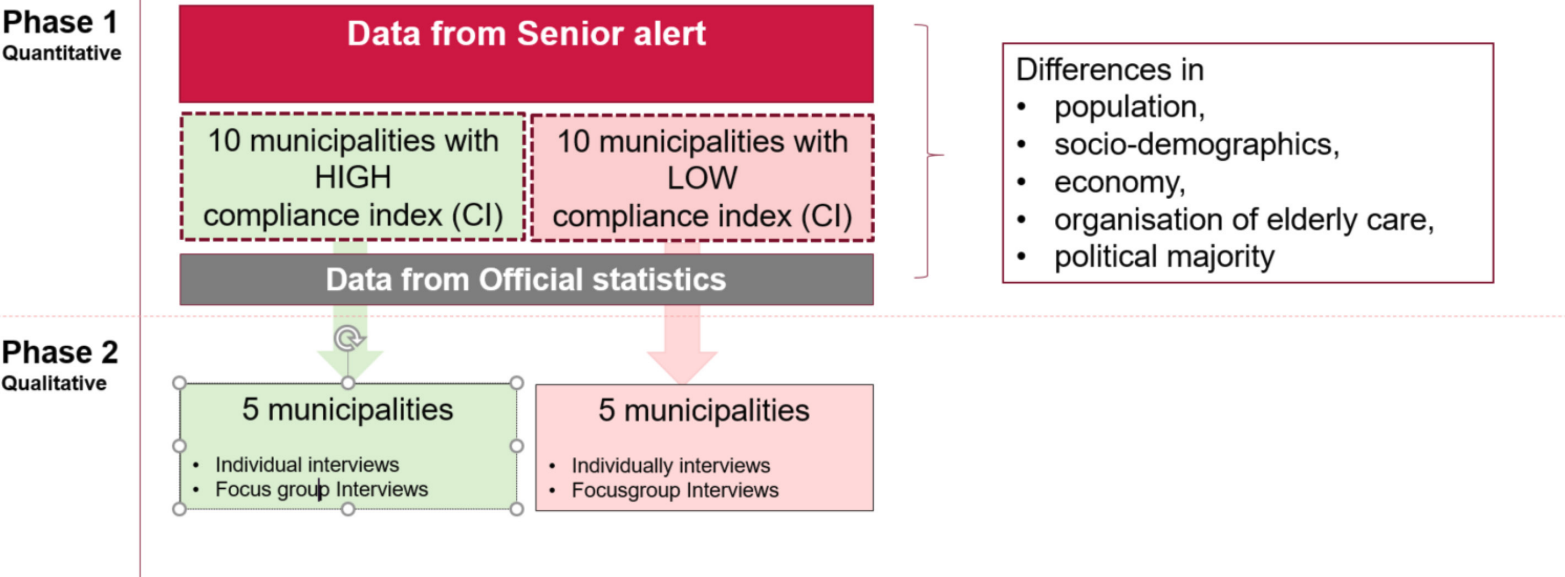
Study design in each phase.

An explanatory mixed methods design^44 45^ divided into three phases will be used (table 1). The timeline is 4 years from 1 November 2021 to 31 December 2025. The focus of this paper is on phases 1 and 2. The first phase is quantitative and will compare municipalities in Sweden using data from official statistics. The second phase is qualitative and will be partly based on the results from phase 1; interviews will be conducted to further explore experiences, preferences and concerns. The third phase involves combining the findings from phases 1 and 2. An improved PcP model will then be designed and tested in a pilot study (figure 3, table 1).

**Table 1:** Mixed methods design in the three phases of the study design

Phases	Focus
1	Quantitative study based on data from SA and official statistics
2	Qualitative study partly based on results from phase 1 to deepen the understanding of collaboration through individual and focus group interviews

### Data collection and participants

#### Phase 1

Data of interest will be extracted from SA. All municipal residential care facilities in all registered municipalities between 2019 and 2021 will be included; however, private municipal residential care facilities and home help care services will be excluded. For each risk assessment, we will check whether the underlying cause, action plan, and follow-up have been registered within the four focus areas: pressure ulcers, weight loss, poor oral health, and falls. All municipalities will receive a compliance index based on their results (table 2). We will select municipalities with a high proportion and a low proportion of complete registrations.

**Table 2:** Compliance index in data selection phase 1

ComplianceIndex	Parts of preventive care process registered
0	Screening for risk assessment
0.33	Screening for risk assessment and one more step in the process
0.67	Screening for risk assessment and two more steps in the process
1	All four steps in preventive care process are completed

Each risk assessment receives a compliance index value (table 2), which constitutes the municipality’s compliance index (compliance indices range from 1=complete registration to 0=totally incomplete registration). For each municipality, we will then calculate the average of these values, which will indicate a compliance factor for each municipality, termed the ‘Compliance’. Subsequently, the 286 municipalities will be sorted according to this indicator. Municipalities with fewer than 1000 risk assessments will be omitted. Among the remaining, the 10 with the highest values of compliance and the 10 with the lowest values of compliance will be studied further. The calculation will be based on the total number of completed registrations and expressed as a percentage in each of the PcP focus areas. Official statistics from SCB (Statistics Sweden) and Kolada (Municipality and County Council Database in Sweden) will be used in the analysis. SCB is the official statistical authority in Sweden. It collects, processes and publishes a comprehensive array of statistics across various domains, including population, economy, labour market, health, education and more, based on data from a variety of channels, including surveys, registries and other administrative data sources in Sweden. Kolada provides information about municipalities and regions and encompasses a range of statistical and financial data that can be used for analysis and comparison between municipalities and regions in Sweden. These data are based on information submitted in various surveys from government agencies, enterprises and private persons. Data will all be continuous, and the values obtained will be expressed as means and SD. Student’s t-test will be performed to compare mean values when the ratio between the greater group SD and the smaller group SD is less than 2, otherwise Welch’s t-test will be used. The statistical analyses will be performed using R V.4.3.0 by the R Foundation for Statistical Computing. All statistical tests will be two-tailed, and levels of significance for p values will be set at<0.10, <0.05 *, <0.01 **, <0.001 ***. To assess how our two groups of municipalities differ from the rest of Sweden, we will conduct the same statistical analysis, comparing low- and high-index municipalities separately with all other municipalities in Sweden, excluding the four largest.

#### Phase 2

The research process begins with the selection of municipalities registered in SA between 2019 and 2021, focusing on those demonstrating both high and low compliance with the preventive process in the areas of pressure ulcers, nutrition, oral health and falls. 10 municipalities, 5 with high and 5 with low compliance, will be chosen based on their proportion of complete registrations in SA. Following this selection, all ten municipalities will be contacted via an email to the most senior manager in elderly care to request participation in the study. After a week, a follow-up will be conducted and reminders sent if necessary. Subsequently, focus group interviews will be conducted in each selected residential care unit, including one with interprofessional teams and another with older persons and/or next of kin. Additionally, 30 semi-structured individual interviews will be conducted with residential care managers at group and organisation levels. In total, between 110 and 170 key informants will be included. The qualitative aspect of the study will explore the importance of leadership for effective care collaboration across organisational boundaries, focusing on identifying obstacles to or opportunities for carrying out Person-Centred Practice. The results obtained in the initial phase will inform the semi-structured individual interviews and the focus group questions, which will centre on obstacles to and opportunities for using SA to achieve a more effective PcP and care collaboration within and/or between teams and different care providers. All interviews will include questions addressing the three attributes considered important for care collaboration: sharing, teamwork and respect. Informed consent will be obtained from all respondents, and basic information regarding age, sex and years in service will be collected during the interviews.

### Patient and public involvement statement

Involving representative older adults and engaging care professionals will be central to the whole research process. We plan to create a dialogue and collaboration with both representative older adults and the general public to jointly design and evaluate methods, processes and outcomes. This includes invitations to annual dialogues to reflect on our activities and how new working models can be integrated into the local context.

### Methodological and theoretical considerations

A strength of this study is the mixed methods design, which will offer the possibility for the examination of the PcP from different perspectives. It will also provide opportunities to gain a deeper understanding of, and important insights into, the complexity of the ***health and social care of older adults.*** In addition, the ambition is to add knowledge about how an implementation strategy for an effective PcP can be developed in elderly care in Sweden. The project will apply the theory of organisational learning^46^ in order to understand how experiences, beliefs and attitudes contribute to the routines and practice of organisations,^47 48^ taking into consideration the institutional complexity of public organisations^49^ and the collaboration between these organisations.^50^ A systematic review of articles describing the implementation of person-centred care for older persons in out-of-hospital settings^51^ showed that interprofessional collaboration, with a focus on preventive measures, is crucial for optimal care practice both for, and in collaboration with, older persons and their relatives, within their own unique circumstances.

## Data analysis

### Phase 1

Data processing and analysis of risk assessments/interventions will be performed regarding the quantitative data. Descriptive and analytical statistics will be used for the analysis.

### Phase 2

The research process will employ a systematic analysis, following Creswell and Poth^44^ for mixed-methods research. Data from various contexts and key informants will be collected and the qualitative content analysis approach, as described by Hsieh and Shannon,^52^ will be employed to identify categories based on the study’s theoretical frameworks. The coding process will be systematic, guided by the attributes of care collaboration, and structured according to the Person-Centred Practice framework and the Theory of Organisational Learning. To enhance the reliability and validity of the findings, several strategies will be implemented. First, multiple coders will be involved in the analysis process where several researchers will in the research group, independently, will review and code the transcripts, which reduces the risk of subjective or biased interpretations and ensures a more objective and balanced analysis. To further support this, a detailed audit trail will be maintained, documenting each step of the coding process, including decisions related to the development of codes and themes. This provides transparency and allows the entire process to be reviewed by the reference group. This external review ensures the objectivity of the findings and helps identify any potential biases or overlooked aspects. Additionally, the analysis will include iterative refinement of codes and themes, enabling the researchers to continually refine and improve the categorisation of the data. Furthermore, the study will validate the findings through member checking, where participants and the reference group will review the results to ensure the accuracy and trustworthiness of the data. By applying these rigorous methods, the research will offer a comprehensive and reliable analysis, contributing to a deeper understanding of the complexities surrounding care collaboration and PcP in the study. These measures will strengthen the study’s credibility, ensuring that the findings are both valid and trustworthy. The analysis will involve coding transcripts systematically, guided by the attributes of care collaboration. To enhance methodological rigour the analysis process will include (1) a holistic understanding of the data, (2) applying a structured coding framework derived from the PcP framework and the Theory of organisational learning and (3) iterative refinement of codes and themes. Finally, the findings will be validated through member checking with participants and the reference group to ensure accuracy and trustworthiness.

## Discussion

The study protocol aims to address the pressing issue of preventive care for older adults, who represent a significant proportion of the population and are among the most vulnerable groups in society. Effective preventive care, coupled with robust care collaboration, is essential for averting adverse events such as malnutrition, poor oral health, pressure ulcers and falls among frail older adults. The insights gained from this study hold the potential to significantly enhance our understanding of how preventive care strategies for older adults can be optimised. To ensure the success of the study, a steering group and a reference group will be established. The steering group will provide an overarching perspective and contribute to the focus and implementation of the project. The reference group will serve as a critical partner, engaging in dialogue with the researchers throughout the project’s duration. Meetings will be held with both groups at least twice a year to provide updates on the substudies and their findings, as well as to plan for future research activities. Furthermore, the input and feedback from these groups will be invaluable for refining and developing the project further. Beyond scientific publication, the dissemination of research findings to stakeholders responsible for the care of older adults within municipalities and regions will be a crucial aspect of the project, ensuring that the knowledge generated is translated into actionable insights that can directly benefit the target population. The explanatory mixed methods study^53^ is based on three phases exploring factors that constitute obstacles to and opportunities for well-functioning care collaboration within and between different care providers. The focus for collaboration is the PcP, based on the quality register SA. Knowledge about how care collaboration works in the context of preventive measures for frail older adults in Sweden is currently limited.^54 55^ The SALAR has launched several initiatives aimed at obtaining such knowledge. The quality registers constitute an important resource in the ambition for the sustainable development of future healthcare where person-centred, locally available and equitable care is applied.^56^ Additionally, they enable structured care information as a basis for follow-up, benchmarking of the quality of care between municipalities and decision-making. Another initiative is the National System for Knowledge-based Management within Swedish Healthcare, the National Programme Groups,^4^ a joint system to deliver more knowledge-based, equitable, and resource-effective care of high-quality, including prevention, treatment and rehabilitation measures. In total, 26 national programme areas have been covered, where the care of older adults constitutes one area. All these initiatives are dependent on care collaboration within and between different care providers. The rationale for this study is to increase knowledge about the factors constituting obstacles to and opportunities for well-functioning care collaboration within and between different care providers. The explanatory mixed methods design will use an explanatory sequential design, where quantitative data constitute a basis for the first research question. Through individual and focus group interviews, the second research question will capture the voices of key informants to gather their views and experiences of being seen and understood in the context. Quantitative and qualitative research designs have their advantages and disadvantages. When a complex phenomenon is studied, both designs might therefore be needed to reach a more complete understanding of the research problem.^57^ We have chosen an explanatory sequential design since the quantitative data obtained in phase 1 could in part contribute to a starting point for the construction of questions to be asked in the interviews in phase 2. Data from these two phases will then form the basis for the development of an effective PcP model that will be tested in a pilot study in one of the residential care facilities identified in the second phase. If the implementation of the model is successful, we aim to proceed with a research proposal for a full-scale study including residential care facilities in various municipalities with different demographics, socio-demographics, economy, organisation of elderly care and political majority.

## Ethics and dissemination

The study obtained ethical approval (reference number 2022-01520-0, Uppsala avdelning 2 medicin). The research group is grounded in both quantitative and qualitative methodologies, with extensive experience in registry research from national quality registers, as well as quantitative and qualitative studies within healthcare. Their extensive collaboration with various healthcare organisations in municipal and regional settings, including government departments and other state-level authorities, further strengthens their approach. The project involves various professional roles to investigate perspectives of barriers and opportunities for care planning collaboration. There is a risk that these groups, particularly older adults and their caregivers, may feel vulnerable during interviews. The research group acknowledges these risks and will strive to establish a trusting atmosphere during recruitment and interviews. Interviews in municipalities with low rates of complete registrations may be particularly sensitive. The project aims therefore to minimise the risk of stigmatisation in the facilities where the interviews take place, given the expectations of both authorities and citizens for high-quality elderly care. Dissemination of the project’s findings will occur through presentations planned at national and international scientific conferences, as well as publications in scientific and popular science journals and websites. Networking presentations commenced in 2022, with the expected publication of results from early 2025 onwards. By disseminating the project findings, the research group aims to stimulate new knowledge and promote the conditions for effective and high-quality elderly care in Sweden.
