# Supplementary material for: Obstacles and opportunities for care collaboration through the utilisation of a preventive care process for frail older adults: a study protocol for a mixed methods study design
Source: BMJ Open. 2025 Feb 10;15(2):e081925. doi: 10.1136/bmjopen-2023-081925 (PMC11815419; doi:10.1136/bmjopen-2023-081925)
Supplement: Supplementary file 1 [file bmjopen-2023-081925.reviewer_comments.pdf]

PEER REVIEW HISTORY

BMJ Open publishes all reviews undertaken for accepted manuscripts. Reviewers are asked to complete a checklist review form and are provided with free text boxes to elaborate on their assessment. These free text comments are reproduced below.

ARTICLE DETAILS

Title (Provisional)

Obstacles to and opportunities for care collaboration through the utilization of an effective preventive care process for frail older adults: A study protocol for a mixed methods study design.

Authors

Boström, Martina Karin; Åsa, Larsson Ranada; Wijk, Helle; Skott, Pia; Erichsen, Anette; Rothenberg, Elisabet

---

VERSION 1 - REVIEW

**Reviewer**	**1**
**Name**	**Teng , Chiao-Hsin**
**Affiliation**	**UNC-Chapel Hill, School of Nursing**
**Date**	**30-Jan-2024**
**COI**	**I have no competing interests**

Thank you for submitting the paper. The concept of this study is interesting, and the figures are straightforward. However, there is significant room for improvement in the writing. The primary issue is a lack of detail. Please see the points below.

Background & Rationale

1. The connection between frailty, the healthcare system, and multidisciplinary collaboration is poorly established.

2. The knowledge gap is unclear. Please link the knowledge gap with current literature and policy challenges in healthcare in Sweden.

3. The necessity of using a mixed-methods design to address the research questions is not evident.

After reading the sections on background and rationale, I am unable to understand the importance of addressing these research questions. That is, the first two sections need reorganization to inform readers about the knowledge gaps and the need for a mixed-methods design.

Methods

1. Based on the phases described - "The first phase is quantitative and will compare municipalities in Sweden using data from official statistics. The second phase is qualitative and will be partly based on the results from phase 1…The third phase involves combining findings from phases 1 and 2. An improved PcP model will be designed and tested in a pilot study" - this design should be characterized as an explanatory mixed-methods design, not convergent. (Creswell & Clark, 2017)

2. The description of statistics for phase 1 is vague. The operational definition of variables (e.g., socio-demographics, economy, and organization of elderly care) and details of statistical methods are missing.

3. In phase 2, how are five units selected out of the 10 municipalities with high/low compliance to be interviewed?

4. It is rare for studies to use both focus groups and individual interviews. Please clarify the rationale/goals for using both approaches in this qualitative part. The authors explained that the focus groups address obstacles to and opportunities for utilizing SA to achieve a more effective PcP and on-care collaboration in and/or between teams and different care providers, but the goal for individual interviews has not been explained.

5. In phase 3, it is unclear how the theory of organizational learning will be incorporated into the development of the PcP program. It is also necessary to briefly introduce this theory to the readers.

Discussion

1. The authors used entirely different terms to describe their study design Convergent, explanatory, and exploratory mixed-methods designs are three different kinds of design.

Reference for mixed-methods methodology

Creswell, J. W., & Clark, V. L. P. (2017). Designing and conducting mixed methods research (3rd ed.). Sage publications.

**Reviewer**	**2**
**Name**	**Gorbenko, Ksenia**
**Affiliation**	**Icahn School of Medicine at Mount Sinai**
**Date**	**19-Mar-2024**
**COI**	**None**

Interesting protocol! Please see some comments to make it compliant with the format plus some clarifying questions.

BMJ Open: Obstacles to and opportunities for care collaboration through the utilization of an effective preventive care process for frail older adults: A study protocol for a mixed methods study design.

Section	Strengths	Limitations	Comment
**Title**	Clear, study population included, design included, clear idea of future paper content (obstacle, opportunities for preventive care in frail older adults)		
**Keywords**			Suggest adding “protocol” to keywords
**Abstract**	Well written and easy to understand	Do not follow the conventional template<br><br>Recommend the following :<br><br>Introduction<br><br>Methods and Analysis<br><br>Ethics and dissemination<br><br>Trial registration (if applicable)	Would recommend<br><br>1/ Follow conventional template<br>2/ Shortening the background section to the essentials, for example removing the first sentence and combining into one sentence this segment “However the way in which, … the process of care collaboration function”
**Strengths and Limitations**		The section is missing	1/ Recommendation to add it based on author’s guideline: “ Please include a ‘Strengths and limitations of this study’ section after the abstract. This section should be no more than 5 bullet points relating specifically to the methods - not the results of the

			study. This will be published as a summary box after the abstract in the final published article.”
**Introduction**	Relevance of the topic<br><br>Aim stated<br><br>Rationale stated<br><br>Research question stated	The structure of the background is not clear.	Questions : Citation for this statement “ From a healthcare perspective, frail older adults probably constitute the largest vulnerable group in society.”<br>? We are asking because in other countries immigrants or individuals with low SES status also are vulnerable groups, is it the case in your context?<br><br>Recommendations:<br><br>1/ Reduced subheading to those recommended in checklist (Introduction with following subheadings<br>- rationale<br>- study aims and hypothesis<br><br>2/ Combine and condense content in background and Governance of the care of older adults in Sweden (explains the rationale/ relevance of the study) to form the introduction<br><br>3/ Senior alert should be move to methodology because it seems to be where the quantitative data will be extracted<br><br>4/ Aim: Recommend adding the desire to develop a model of care prevention as stated in background<br><br>Your interviews will include local politicians, older individuals and next of kin as well, we recommend changing healthcare professionals to maybe stakeholders.<br><br>5/ Suggest using the word “registry” in place of “register.”

			Please correct the following typos: semi-structured (p. 2), caregivers (p. 5), healthcare system (instead of “healthcare production” on p. 7)
**Methodology**			Questions:<br>Could you explain the difference between population characteristics and socio-demographics<br>?<br><br>Suggestions:<br>1/ For phase 2, the FINCA conceptual framework could be a reference to consider when drafting the interview guide (https://www.ncbi.nlm.nih.gov/pmc/articles/PMC10577300/)<br><br>2/ The manuscript refers to both “convergent mixed methods design” and “explanatory sequential research design.” It seems that the latter is applicable because the quantitative part of the study (Phase 1) is the basis for the qualitative part (Phase 2) like the authors explain on p. 15, line 28 and below. It is not clear to me what you mean by “The convergent mixed methods design will use an explanatory sequential design”<br><br>3/ In Phase 2, please indicate how you will screen older adults for participation (e.g. absence of cognitive decline, willingness to participate) and if there are any incentives planned for participants

**Ethic and dissemination section**		Section is missing	Recommendation:<br><br>1/Recommend adding based on author's guideline: Ethics and dissemination: ethical and safety considerations and any dissemination plan (publications, data deposition and curation) should be covered here.
**Conclusion**		The study protocol should not include any conclusion (BMJ open requirements)	Recommend Removing conclusion
**References**	Recent (less than		

	10yrs)		

VERSION 1 - AUTHOR RESPONSE

We have considered all Your feedback and have now made the revisions You suggest. We believe that these changes have only strengthened our manuscript and we are very greatful for all Your comments, thank You! Overall we have made the following major revisions (A more detailed response to each comment is included in the attached document, ensuring that all reviewer comments were thoroughly addressed.

**Reviewers comment**	**Response from resarcher team**
Background & Rationale<br>1. The connection between frailty, the healthcare system, and multidisciplinary collaboration is poorly established.<br>2. The knowledge gap is unclear. Please link the knowledge gap with current literature and policy challenges in healthcare in Sweden.<br>3. The necessity of using a mixed-methods design to address the research questions is not evident. After reading the sections on background and rationale, I am unable to understand the importance of addressing these research questions. That is, the first two sections need reorganization to inform readers about the knowledge gaps and the need for a mixed-methods design.	Yes, we agree - the lack of knowledge about the connection between frailty, the healthcare system, and multidisciplinary collaboration is actually an urgent problem for the society and we have tried to clarify this in the manuscript.<br><br>We will use convergent mixed-methods analysis since we seek to understand both the outcomes and processes, patterns, and individual experiences within their study. We will interview (se manus) to ensure that we include all relevant organisations, stakeholders, managers and health-care personel.<br><br>Doing this the study will provide a richer, more nuanced perspective on the research problem, ultimately leading to more informed and effective conclusions and recommendations.
Methods<br>1. Based on the phases described - "The first phase is quantitative and will compare municipalities in Sweden using data from official statistics. The second phase is qualitative and will be partly based on the results from phase 1…The third phase involves combining findings from phases 1 and 2. An improved PcP model will be designed and tested in a pilot study" - this design should be characterized as an explanatory mixed-methods design, not convergent. (Creswell & Clark, 2017)<br><br>2. The description of statistics for phase 1 is vague. The operational definition of variables (e.g., socio-demographics, economy, and organization of elderly care) and details of statistical methods are missing.<br><br>3. In phase 2, how are five units selected out of	1. Thank You for this important methodological comment, we agree and have updated the manuscript.<br><br>2. Thank You – we have now added details of statistical methods.<br><br>3. We have revised the text to clarify how the five units are selected from the 10 municipalities with high/low compliance for interviews in phase 2.<br><br>4. We use both individual interviews and focus group interviews in our mixed study design to gain a more comprehensive perspective from multiple individuals. By combining in-depth interviews with focus groups, we aim to enhance the credibility and diversity of our results.<br><br>5. We have revised the description in phase 3 to emphasize that this sub-study will build upon the

the 10 municipalities with high/low compliance to be interviewed?<br><br>4. It is rare for studies to use both focus groups and individual interviews. Please clarify the rationale/goals for using both approaches in this qualitative part. The authors explained that the focus groups address obstacles to and opportunities for utilizing SA to achieve a more effective PcP and on-care collaboration in and/or between teams and different care providers, but the goal for individual interviews has not been explained.<br><br>5. In phase 3, it is unclear how the theory of organizational learning will be incorporated into the development of the PcP program. It is also necessary to briefly introduce this theory to the readers.	results from phases 1 and 2. Due to the foundational nature of patient involvement in our approach, we find it challenging to provide a detailed account of how this will be executed. We appreciate your understanding of our methodology.
Discussion<br>1. The authors used entirely different terms to describe their study design Convergent, explanatory, and exploratory mixed-methods designs are three different kinds of design.	We have updated the terminology for our study design; it is now solely an explanatory mixed-methods design.
References	We have updated all references in the reference list.

VERSION 2 - REVIEW

**Reviewer**	**1**
**Name**	**Teng , Chiao-Hsin**
**Affiliation**	**UNC-Chapel Hill, School of Nursing**
**Date**	**18-Nov-2024**
**COI**	

Thank you for the revision. However, there are several major issues that remain unresolved, as outlined below.

Major Comments:

1. Background and Rationale

The background section remains vague, making it difficult to fully grasp the rationale behind the chosen methodologies. The constructs of care collaboration, frailty, and utilization of preventive care processes and their relationships are not clearly defined.

For instance, after reading the background, it is still unclear what specific healthcare problem exists in Sweden and why compliance is being investigated. Moreover, how does this compliance relate to organizational collaboration?

On pages 3 and 9, I strongly recommend the authors rewrite the paragraphs to integrate context (e.g., Senior Alert and PcP) into the introduction. This will help clarify how the study leverages these systems to address the identified issue of insufficient collaboration.

2. Mixed-Methods Design

The description of the mixed-methods design is inconsistent between the manuscript and the response letter. In the response letter, it is described as a convergent design, whereas the manuscript refers to it as explanatory. Please reconcile this discrepancy and ensure consistency.

3. Data Analysis

The explanation of the data analysis approach is insufficient. Content analysis, for instance, has multiple variations. Please specify the type of content analysis being used and cite the relevant methodology to provide clarity and rigor.

4. Phase 3 Co-Design

Phase 3, described as co-design, is not introduced in the early sections of the manuscript or the methods. It first appears on page 14. This creates confusion about whether the study comprises two or three phases. Please address this inconsistency.

5. Statistical Analysis

The manuscript lacks details on the statistical methods used to compare the two groups. Please include this information in the statistical analysis section for clarity.

6. Explanatory Mixed-Methods Design

According to Creswell et al., explanatory mixed-methods analysis should include a third component that explains how qualitative data elucidates quantitative findings to answer the mixed-methods question. Please describe how this integrative analysis will be conducted.

7. Rigor of Qualitative Analysis

The rigor of the qualitative analysis is insufficiently addressed. Collecting individual and focus group interviews alone does not guarantee comprehensive understanding. I recommend referring to Guba’s criteria for enhancing qualitative rigor, such as using multiple coders, maintaining an audit trail, and incorporating external checks. These measures should be explicitly described.

Minor Comments:

1. The statement: “From a healthcare perspective, frail older adults probably constitute the largest vulnerable group in society” is overly general and not scientifically rigorous. I suggest including statistics to substantiate the importance of frailty as a research topic.

VERSION 2 - AUTHOR RESPONSE

Reviewer:

1. Background and Rationale
The background section remains vague, making it difficult to fully grasp the rationale behind the chosen methodologies. The constructs of care collaboration, frailty, and utilization of preventive care processes and their relationships are not clearly defined.

Our respond: We will stress this paper is a study protocol, meaning that we do not yet have any result. We intend to investigate if, why, and how collaboration is crucial for managing the preventive care process in Senior alert for frail older adults. Therefore, the background in this paper cannot yet describe how these elements are related to each other, as this is one of our research questions in the upcoming study . For clarification in the Background frailty is defined as “… a clinical state in which there is an increase in an individual’s vulnerability for developing increased dependency and/or mortality when exposed to a stressor.” *J Am Med Dir Assoc*. 2013 June ; 14(6): 392–397. doi:10.1016/j.jamda.2013.03.022. Furthermore, we have now tried to clarify the relationship between care collaboration, frailty, and utilization of preventive care processes.

2. For instance, after reading the background, it is still unclear what specific healthcare problem exists in Sweden and why compliance is being investigated. Moreover, how does this compliance relate to organizational collaboration?

As stated above paper is a study protocol for a long-term project aiming to explore factors that constitute obstacles and opportunities for collaboration within and between different care providers in utilizing a preventive care process for frail older adults. We have tried to clarify common health problems in the group frail older adults, please see 1) comments above. Furthermore, compliance in the context means compliance to all steps in the PcP, which requires cooperation between different health care professionals and organisations which has now been clarified. To enhance the clarity and accessibility of the rationale behind the study, we have added a dedicated section that explains the reasons for conducting this research

Additionally, we have included the following text at the end of the background section, just before the rationale, to provide further context and support for the study's objectives: ‘In summary, despite prioritized initiatives for preventive care for frail older adults in Sweden, evaluation and follow-up of these processes are not carried out to the extent necessary to assess expected outcomes. This can be linked to insufficient adherence to the process and a lack of collaboration in how care is organized. Adherence to preventive efforts, however, depends on well-functioning collaboration between different care levels and professions, as risk assessments and interventions often require shared responsibility and coordinated actions. Understanding how adherence to preventive care can be improved through effective collaboration may help reduce adverse events and improve the quality of care for frail older adults.'"

3 On pages 3 and 9, I strongly recommend the authors rewrite the paragraphs to integrate context (e.g., Senior Alert and PcP) into the introduction. This will help clarify how the study leverages these systems to address the identified issue of insufficient collaboration.

We have now introduced SA at page 3.

4. Mixed-Methods Design
The description of the mixed-methods design is inconsistent between the manuscript and the response letter. In the response letter, it is described as a convergent design, whereas the manuscript refers to it as explanatory. Please reconcile this discrepancy and ensure consistency.

Thanks for this comment. We are very sorry for this mistake, we have now clarified in the text that we will use the explanatory design further on.

5. Data Analysis
The explanation of the data analysis approach is insufficient. Content analysis, for instance, has multiple variations. Please specify the type of content analysis being used and cite the relevant methodology to provide clarity and rigor.

We have clarified the text by incorporating the following additions into the manuscript. Furthermore, this section has been revised based on the feedback provided by the reviewers during the previous round of revisions. Additionally, we have included the following text to provide further explanation:

”Data from various contexts and key informants will be collected. A qualitative content analysis approach, as described by Hsieh and Shannon (Hsieh HF, Shannon SE. Three approaches to qualitative content analysis. Qual Health Res 2005;15(9):1277-88), will be employed, enabling the identification of categories based on theoretical frameworks of the study. The analysis will involve coding transcripts systematically, guided by the attributes of care collaboration. To enhance methodological rigor the analysis process will include (1 a holistic understanding of the data (2) applying a structured coding framework derived from the PcP framework and the Theory of organisational learning, and (3) iterative refinement of codes and themes. Findings will be validated through member checking with participants and the reference group to ensure accuracy and trustworthiness”.

6. Phase 3 Co-Design
Phase 3, described as co-design, is not introduced in the early sections of the manuscript or the methods. It first appears on page 14. This creates confusion about whether the study comprises two or three phases. Please address this inconsistency.

We sincerely apologize for this oversight. Phase 3 was excluded from the study design in our earlier version; however, this specific section was mistakenly not removed. We have now corrected this error and removed the text accordingly.

***7. Elisabet**: Statistical Analysis*
The manuscript lacks details on the statistical methods used to compare the two groups. Please include this information in the statistical analysis section for clarity.

Many thanks, we have now included this information in the paragraph Statistical Analysis

8. Explanatory Mixed-Methods Design
According to Creswell et al., explanatory mixed-methods analysis should include a third component that explains how qualitative data elucidates quantitative findings to answer the mixed-methods question. Please describe how this integrative analysis will be conducted.

This is correct, and the third component, which involves integrating the results from the two phases to clarify how they interact and contribute to a comprehensive understanding, will be part of the overall project. However, this will not be conducted within the framework of this study protocol. Instead, it will be carried out as a sub-study, which will be funded under a new project.

9. Rigor of Qualitative Analysis
The rigor of the qualitative analysis is insufficiently addressed. Collecting individual and focus group interviews alone does not guarantee comprehensive understanding. I recommend referring to Guba's criteria for enhancing qualitative rigor, such as using multiple coders, maintaining an audit trail, and incorporating external checks. These measures should be explicitly described.

We have consciously chosen to include a more general description of the different parts of our study protocol, in order to let our data guide the choice of method and the execution of the analysis. To strengthen and clarify the methodological rigor of the qualitative analysis, we have expanded the description of the analysis steps in our manuscript acccording to your kind suggestions.

10. The statement: "From a healthcare perspective, frail older adults probably constitute the largest vulnerable group in society" is overly general and not scientifically rigorous. I suggest including statistics to substantiate the importance of frailty as a research topic.

Thanks, we have now clarified this in the text and added the references we have used.

11. If you have selected 'Yes' above, please provide details of any competing interests.: Not applicable

We are not sure we fully understand the comment, but we would like to clarify that we have nothing to declare regarding any competing interests.
